# Sugar Wars: Formulators of Endurance Fuels Compete to Pack More Energy in Fewer Molecules

**DOI:** 10.1021/acscentsci.4c00662

**Published:** 2024-05-02

**Authors:** Robin Donovan

Triathlete Gwen Jorgensen fueled her 2016 Olympic gold with Red Bull, a potent, fizzy mix of sugar, taurine, and caffeine that tastes a bit like cherry cough syrup. Perhaps surprising to non-Olympians, sugar is the most important fuel in that concoction. Athletes rely on carbohydrates, often in the form of sugars, to provide energy and prevent them from “bonking”—or hitting a wall of exhaustion. Glucose is the body’s primary energy source, so athletes look for drinks and gels that will impart a lot of this simple sugar.

Red Bull provides simple sugars like sucrose, which contains a single unit each of glucose and fructose. While that was enough to power Jorgensen to an Olympic medal, she later switched from triathlon to running full time, and longer run sessions meant she needed even more midworkout carbs. Simply gulping down sweet drinks made Jorgensen’s stomach cramp—she needed something that would give her more glucose without upping the concentration of sugary carbs she was taking in.

“Our stomach’s kind of picky,” says Melissa Markofski, an exercise physiologist at the University of Houston. It can generally tolerate only a 6% to 8% concentration of carbohydrates by weight without causing gastrointestinal (GI) issues—often nausea and vomiting. This limitation leaves sports scientists scrambling for ways to make more glucose available during exercise. One mechanism is swapping simple sugars for more complex carbohydrates like maltodextrin, which contains up to 17 glucose units per molecule. So without increasing the concentration of molecules in a drink or gel, more glucose is available with complex carbohydrates.

**Figure:**
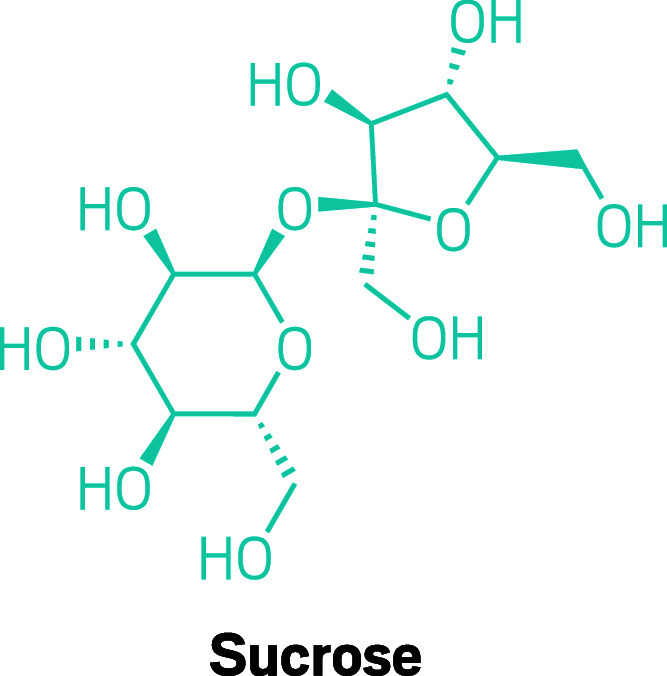
Sucrose, or table sugar, contains a single unit each of glucose and fructose, two simple sugars.

“Broadly speaking, if you’re able to get in more carbohydrates, then you can have a performance advantage,” Markofski adds. But there’s more to it than science can predict. “There’s a reason why all these sports beverages exist—and that is because people *do* have preferences,” she says.

To address her energy and GI needs, Jorgensen tapped physiologist Allen Lim at Skratch Labs, who developed a high-carb mix based on a lesser-known carbohydrate: highly branched cyclic dextrin. The chunky, helical carb contains even more glucose units than maltodextrin—60 to 70 per molecule. Since each molecule can be broken down into multiple units of glucose, scientists hope an athlete’s gut can absorb more glucose units without risking GI distress by increasing the molecular concentration in a drink, gel, or chew.

Two kids and several years later, Jorgensen is back to competing in triathlons and still drinks the mix Lim developed. Her race fuel is a bottle with four scoops of the drink powder and a scoop of an electrolyte-sugar blend. The mixture clocks in at nearly 80 g of carbohydrates, more than two cans of Red Bull—or about as much as 45 Haribo gummy bears, a massive increase from her prior fueling plan.

Sports dietitians’ current advice mirrors Jorgensen’s experience: endurance athletes—even amateurs—need more carbs during longer workouts. And the array of fueling options has exploded to meet that demand. But questions remain about whether the average body can digest and absorb the flood of glucose from complex carbs fast enough to matter, or if the flood of fuel just piles up in the gut. That unknown, alongside steep price tags, cloak-and-dagger research, marketing hype, and the inherent variation in people’s physiology and personal preferences, has prevented the emergence of a clear front-runner—at least for now.

**Figure:**
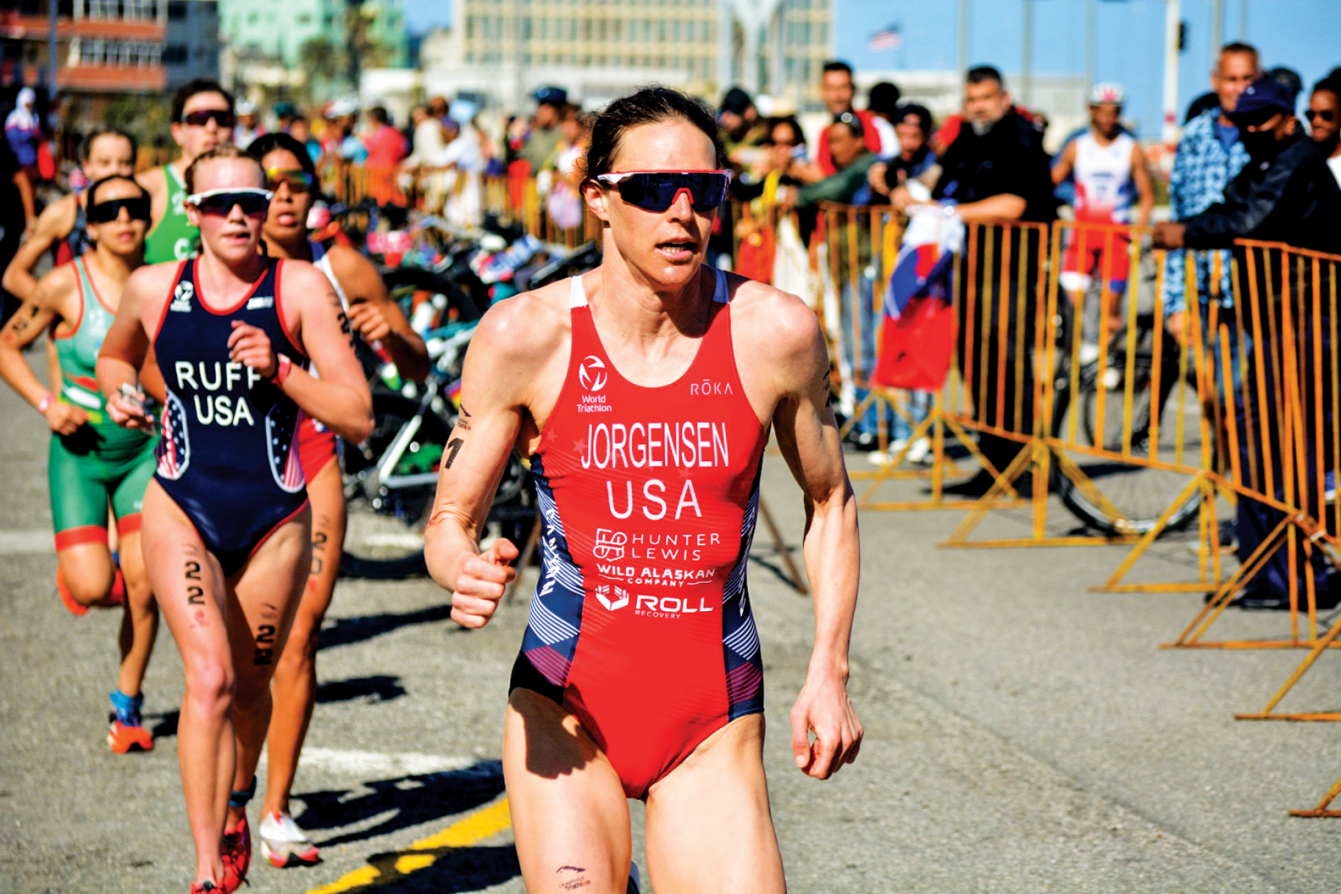
Professional athlete Gwen Jorgensen has switched to a high-carbohydrate sports drink mix that contains highly branched cyclic dextrin, which is less sweet and contains more glucose units than simple sugars. Credit: Courtesy of Gwen Jorgensen.

## Not-so-simple sugars

Some of the science is simple. A human being can typically absorb a maximum of 60 g of glucose per hour—but we can absorb 30 g of fructose per hour at the same time.

Fructose is ultimately converted to glucose in the small intestine, so ingesting both of these simple sugars at once increases the total carbohydrates absorbed to 90 g per hour. (Along with electrolytes, these two carbohydrates are the primary ingredients in Gatorade.) Over the years, hourly carb recommendations have crept from 30 g to 60–90 g, with some professional athletes now consuming 120 g or more per hour.

**Figure:**
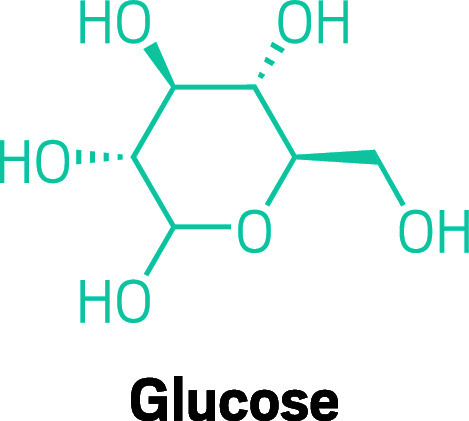
The body’s main source of energy, glucose is the simple sugar our bodies need to power through workouts.

Glucose and fructose dominate sports nutrition. For athletes, upping the type and complexity of carbohydrates can increase the fuel available for workouts. But larger molecules like maltodextrin and highly branched cyclic dextrin may be relatively slow to break down in the intestine. So, it is important to balance the carbs we absorb the fastest with the potential for GI distress, which happens when too much fuel sits in the stomach or intestines—akin to the feeling of needing to loosen your belt after an oversized meal. The additional stress of exercise slows digestion by diverting blood to the muscles and lungs.

Factor in individual physiology and the number of available endurance fuel options, and you understand why endurance athletes become one-person laboratories testing different products. Each competitor wants peak performance from a palatable fuel at a decent price.

## The price of high performance

When it comes to choosing a product, “sometimes people just gravitate towards that more from a branding and marketing perspective than it being like a better product,” says Caitlin Goodman, formerly a sports dietician for the University of Oregon’s track and field team. “There are definitely products that are perceived as a little bit more natural or like real food.”

The Skratch Laboratories’ Hydration Sport Drink Mix is a sucrose-dextrose blend that costs about $22 per pound or roughly 20 servings. (Dextrose is another name for glucose.) The company uses a small amount of real fruit—think lime juice or lemon oil—then adds calcium, potassium, magnesium, and 100 mg more sodium than Gatorade’s Endurance Formula per serving.

Jorgensen mixes the hydration powder with Skratch’s Super High-Carb mix, which contains highly branched cyclic dextrin and costs about the same by weight, though its larger serving size means that 8 servings cost nearly $42. By contrast, Gatorade products with simple carbs start at $2.75 per pound on Amazon, which is also about 20 servings. Gatorade’s souped-up drink mix with additional electrolytes costs about $11.50 per pound—about 19 servings.

Maltodextrin is a favorite of budget-minded athletes who handle their hydration at home, often mixing the complex carb with salt tablets and fructose to mimic commercial products. Among professional athletes, Skratch Laboratories sponsored Jorgensen in the past and filmed her testing the product; she is not currently sponsored.

Sports fuel purveyor Maurten sells gels that are firm, glucose/fructose-based, and cost about $4–5 per serving, depending on the amount of carbs. That cost means an amateur cyclist on a lengthy ride could go through $20 in gels alone. Maurten’s maltodextrin-based drink mixes are up to $3 per serving. Yet the products are offered as race-day calories at Ironman events around the globe. Some amateur athletes buy them for training, hoping to simulate course conditions during sessions at home. Maurten has featured Kenyan marathoner Eliud Kipchoge in its marketing. He told the company that its drink mix in its early years was too sweet; the company adjusted the blend, and Kipchoge has since used it to set multiple world records.

**Figure:**
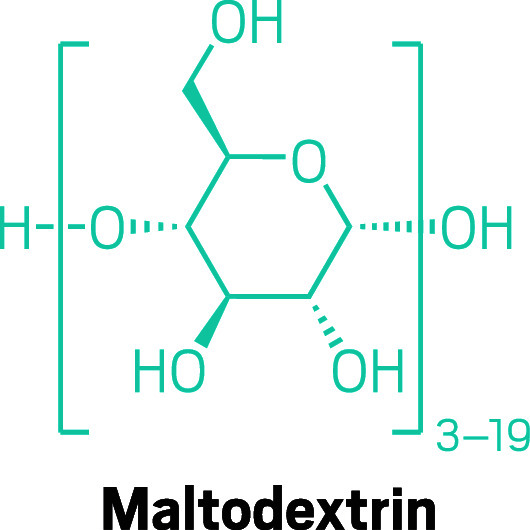
A popular, inexpensive, complex carbohydrate, maltodextrin is lightly sweet yet offers more glucose units per molecule than the simple sugars prevalent in products such as Gatorade.

Markofski says the progression from fuels dominated by glucose to maltodextrin to highly branched cyclic dextrin is logical. Glucose-fructose mixes are cheap but taste very sweet. Maltodextrin, an inexpensive starch, is a more complex carbohydrate with much less sweetness. Highly branched cyclic dextrin has an even more complex structure with additional units of glucose on a helix-shaped, glob-like molecule—and has the same barely there sweetness as maltodextrin. With the success of maltodextrin in sports drinks, “It kind of makes sense, then, that we would want to find something that works even better,” Markofski says.

## Do complex carbs really work?

Manufacturers claim that highly branched cyclic dextrin works, but it is not clear what length or type of exercise it was tested on or whether, for example, recreational athletes were included in their studies. “A lot of times, these companies do their own research, but it doesn’t get published or peer reviewed,” says Markofski.

**Figure:**
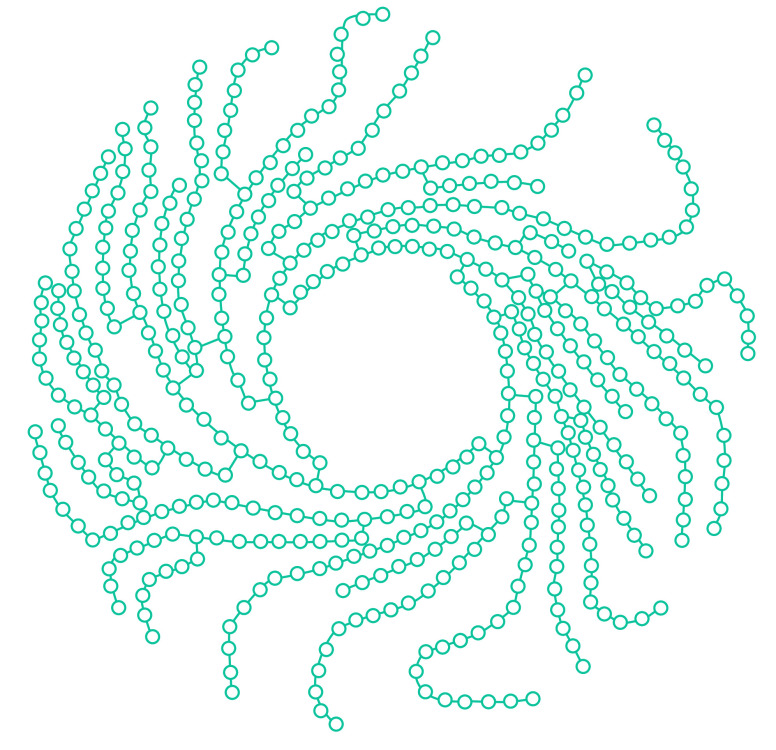
A schematic representation of highly branched cyclic dextrin, in which each circle represents a glucose unit. Endurance athletes are flocking to products with highly branched cyclic dextrin, which can contain up to 70 units of glucose per molecule. Credit: Adapted from *Eur. J. Pharm. Sci.*

A key claim for highly branched cyclic dextrin, which also goes by the brand name Cluster Dextrin, is that it empties from the stomach faster than glucose, potentially preventing bloating and cramping.

What happens to those glucose-packed molecules next is unclear. It seems plausible to exercise physiologist Dan Baur that larger molecules would break down more slowly in the gut. “The intestinal absorption rate just sort of is what it is,” says Baur, who works for the Virginia Military Institute. “So, it doesn’t matter how quickly you get it to the intestines. They are only capable of taking up a certain amount of carbohydrate in a certain amount of time.”

Baur is not convinced that slower digestion benefits endurance athletes. Neither is sports dietician and endurance expert Alex Larson, although a number of the athletes she advises swear by highly branched cyclic dextrin products. “The product itself has some claims that it can reduce GI issues,” she says. “I don’t think it necessarily can do that for everyone just because, with GI issues, there are so many variables.”

Describing highly branched cyclic dextrin as a “globule,” Baur says the product delivers more total grams of carbs for the same osmolarity, or molecular concentration, than glucose, fructose, or maltodextrin. He adds, backing Larson’s view, that the larger molecules, once they reach the intestine, “might actually sort of gunk it up and slow it down at that point.” In other words, cramming more glucose units into the same number of molecules may create its own consequences.

David Rowlands is an expert in exercise metabolism at New Zealand’s Massey University whose research provided early validation for the now-standard 1:0.8 ratio of glucose to fructose in 2013. He has a practical opinion shared by most experts quoted here: highly branched cyclic dextrin’s main benefit is that it empties quickly from the stomach, limiting the chance of cramping and GI distress. However, its high cost and the uncertainty of intestinal absorption mean that for amateur athletes, the time to switch is after exhausting cheaper options.

Erika Hamel, a distance runner from New Hampshire, uses a highly branched cyclic dextrin product for runs over 15 miles. Her stomach can’t tolerate chews, and the texture of gels nauseates her, so she can barely eat them during runs. “I was very excited about getting my calories easily from a drink mix,” she says.

Rowlands says only professional athletes, for whom scraping seconds from a marathon time is meaningful, or amateurs who get queasy like Hamel, who’d otherwise spend race day in a port-a-potty, are likely to benefit significantly from highly branched cyclic dextrin.

**Figure:**
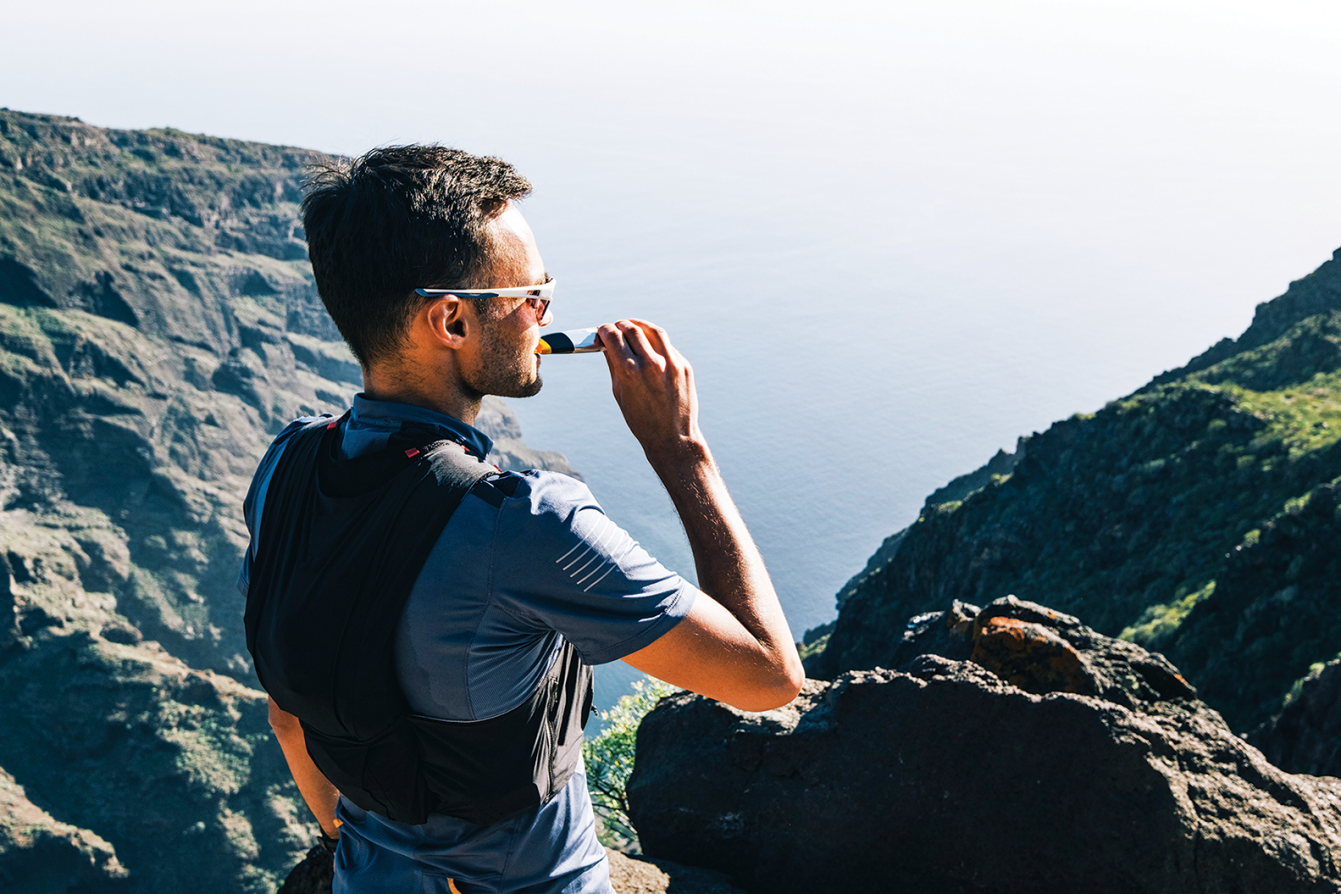
Endurance athletes often fuel longer workouts using packets of viscous, sugary gels. Typically, each contains enough carbs for 30 to 60 min of exercise. Credit: Shutterstock.

In her car between workouts, Jorgensen is prepping for a cycling workout with 40 high-intensity efforts. She’ll use a motorcycle for pacing, gulping down a bottle with two scoops of Skratch’s high-carb mix and Hydration blend during the hour-long session. She’s hardly concerned that the science on highly branched cyclic dextrin is uncertain.

“I’m very comfortable just knowing that what I use works for me, gives me enough calories, and doesn’t cause me any GI distress,” she says. “Even if science proves something, it doesn’t mean that it’s going to work for everyone.”

*Robin Donovan is a freelance contributor to*Chemical & Engineering News*, the independent news outlet of the American Chemical Society.*

